# Nickel Challenge In Vitro Affects CD38 and HLA-DR Expression in T Cell Subpopulations from the Blood of Patients with Nickel Allergy

**DOI:** 10.3390/ijms25010298

**Published:** 2023-12-25

**Authors:** Metin Artuc, Torsten Zuberbier, Matthias Peiser

**Affiliations:** 1 Department of Dermatology and Allergy, Allergy Center Charité, Charité-Universitätsmedizin Berlin, 10117 Berlin, Germany; 2 Berlin Institute of Allergology, Charité-Universitätsmedizin, Campus Benjamin Franklin, 12203 Berlin, Germany; torsten.zuberbier@charite.de; 3 Fraunhofer Institute for Translational Medicine and Pharmacology ITMP, Allergology and Immunology, 12203 Berlin, Germany; 4 Institute for Chemistry and Biochemistry, Free University Berlin, 14195 Berlin, Germany

**Keywords:** nickel allergy, lymphocytes, cytotoxic T cells, T helper cells, phenotyping, flow cytometry

## Abstract

Nickel allergy is a major health problem and shows clinical manifestation of contact eczema. The response of specific lymphocyte subpopulations in sensitized patients after new challenge to nickel has until now not been studied in detail. To evaluate if nickel-based elicitation reaction could be objectively identified by multi-parametric flow cytometry, immunophenotyping of specific T cells was applied. White blood cells from 7 patients (4 positive in patch test, 3 negative) were challenged by nickel and in vitro short-term culture. Standardized antibody-dye combinations, specific for T helper(h)1, Th17 and cytotoxic T cell activation, were selected according to the recommendations of Stanford Human Immune Monitoring Center. In cytotoxic CD8^+^CCR7^+^CD45RA^+^ T cells from patients suffering from nickel allergy, CD38 and HLA-DR were elevated comparing to healthy donors. After challenge to nickel in vitro both markers decreased in CD8^+^CCR7^+^CD45RA^+^ T cells but found up-regulated in CD4^+^CCR7^+^CD45RA^+^CCR6^−^CXCR3^+^Th1 cells. Intracellular expression of T-bet and RORγt further indicated Th1 and Th17 cells. Finally, CD4^+^CD25^+^CCR4^−^ T cells increased after challenge with nickel in PBMCs of patients with nickel allergy. Flow cytometry based quantification of T cell markers might be used as a specific and reliable method to detect chemical induced skin sensitization and confirm diagnostic patch testing in the clinics.

## 1. Introduction

Immunophenotyping using flow cytometry has become a reliable technique in a broad range of clinical applications such as biomarker-based diagnosis and prognosis in oncology and COVID-19. However, it is only sparsely used in clinical detection of human allergies. This is surprising as a complete panel of biomolecules associated with inflammatory processes specific for food allergy and allergic contact dermatitis (ACD) are reported [1,2,3]. Observed in inflammatory reactions different from allergies, activation markers of dendritic cells and T cells are found to be modulated in type 1 and type 4 reactions as well. In addition to the potential increase of expression level for such inflammation marker on an individual immune cell population, T helper (Th)cell subpopulations such as Th1, Th2 and Th17 emerge and shift after antigen or allergen exposure [4,5].

To standardize T cell immunophenotyping in globally distributed flow laboratories, the Stanford Human Immune Monitoring Center and others published recommendations [6,7,8]. A common feature of the different approaches is besides the choice of antibodies and fluorochromes or lanthanide metals (CyTOF) used, the definition of a panel of specific and agreed markers expressed on different subpopulations and corresponding to their level of activation. Th1 cells for instance, are according to the protocol characterized by expression of the markers CD4, CCR7, CD45RA, CXCR3, but not by CCR6. 

Th cells are mainly known to play a crucial role in response to pathogenic bacteria and worms [4,5]. Moreover, Th cells are supposed to be involved in ACD as well [9,10,11,12]. For characterization of chronic immunodeficiencies these FACS methods detecting T cells are proposed by different authors [13,14]. However, for use as a diagnostic tool of inflammatory ACD or food allergy, there is no comprehensive method reported in the literature. For that reason, we questioned if surface molecules on human T cells could be used as reliable diagnostic markers for confirmatory identification of ACD. In this proof-of-principle approach, we observed changes in expression of functional surface molecules in patients suffering from nickel allergy after challenge in vitro and compared the expression pattern to healthy donors.

The metal nickel is an environmental contact allergen with high impact on human health because it is used in products associated with potential rupture of skin barrier such as jewelry and piercings. Nickel-specific CD8^+^ T cytotoxic cells, but also CD4^+^Th1 and Th2 from patients with eczematous dermatitis demonstrated strong MHC-restricted cytotoxicity against target cells coupled to nickel [15]. If stimulated in vitro by nickel, CD4^+^ T cells from allergic and nonallergic subjects undergo proliferation [16]. In contrast, nickel-specific proliferation of CD8^+^ T cells was observed in allergic patients but not healthy individuals. In the latter group, MHC class II (HLA-DR) molecules could play a crucial role in nickel-induced T cell proliferation [17]. On the other side nickel affects the T cell receptor by an individual binding mechanism of nickel ions to coordination sites on HLA-DR/peptide conjugates [18,19]. Nickel was further shown to trigger pathogen receptor TLR4 by direct interaction, providing thereby an additional inflammatory signal [20]. With respect to T cell polarization, nickel affects immune-inhibitory molecules cooperating between dendritic cells and T cells. Nickel-dependent modulation of cytokine release from Th cells via programmed death-ligand 1 was recently shown by our group [21]. In peripheral blood mononuclear cells (PBMCs) from human blood, nickel induced memory CD40 ligand positive Th cells expressing TCR α-chain segment TRAV9-2 [22]. 

## 2. Results

### 2.1. Identification of CD3^+^CD8^+^CCR7^+^ T Cells in Blood from Nickel Sensitized Patients

**Figure 1:**
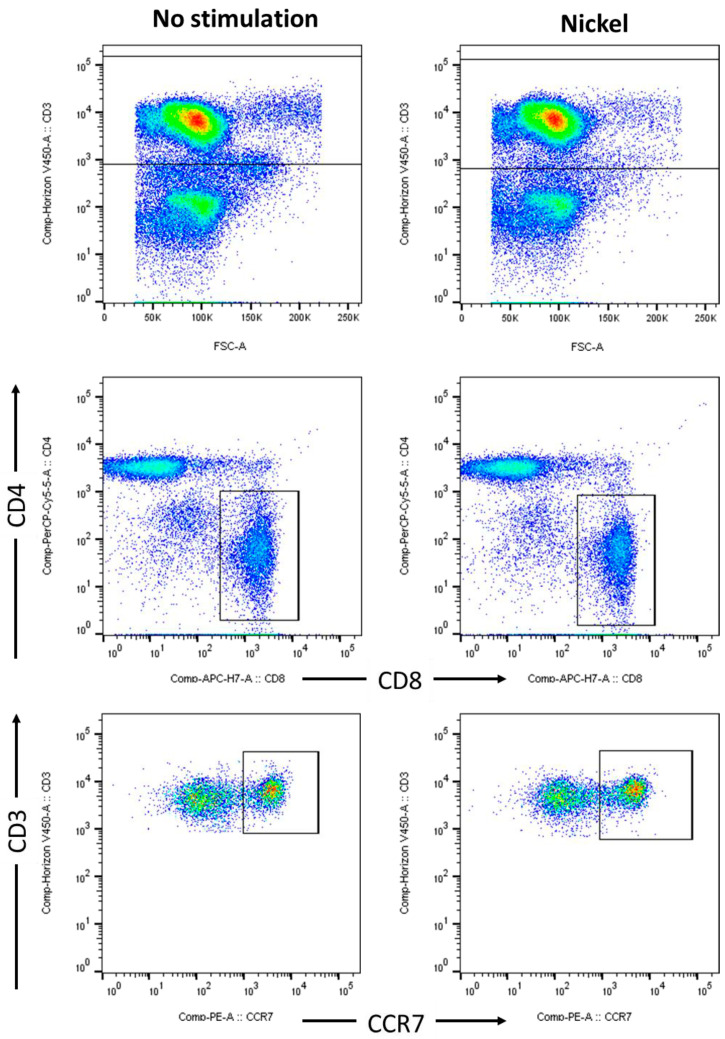
Cytotoxic T cells: Gating strategy and immunophenotyping of nickel re-stimulated PBMCs from nickel allergic patients. Flow cytometric analyses of PBMCs from human peripheral blood, stimulated for 48 h by 200 µM NiSO_4_ (**right** panel) or control (**left** panel). According to the recommendations of Stanford Human Immune Monitoring Center, naïve and central memory T cells were stained and discriminated by CD3 (**upper** row), CD4 vs CD8 (**middle** row) and CCR7 (**lower** row). FACS analyses show dot plots of one donor, representing results from a total of n = 4 patients suffering from nickel allergy; PBMC, peripheral blood mononuclear cells, NiSO_4_, nickel(II)sulfate.

ACD was confirmed by diagnostic patch testing in four patients suffering from nickel allergy at Department of Dermatology and Allergy, Allergy Center Charité. In parallel to clinical testing according to guidelines of European Society of Contact Dermatitis [23], PBMCs were prepared from blood samples of the patients. PBMCs were re-stimulated by nickel in vitro cell culture and analysed by multi-parametric flow cytometry. Therefore, immunophenotyping was performed according to the recommendations of Stanford Human Immune Monitoring Center. Naïve and central memory cytotoxic T cells were discriminated by expression of CD3, CD8 and CCR7 (Figure 1). Standard values for % positive cells and MFI were received for the lineage markers such as CD4 and CD8 in normal PBMCs. 

### 2.2. CD38 and HLA-DR in PBMC from Patients with Nickel Allergy Diminish after Nickel Challenge

**Figure 2:**
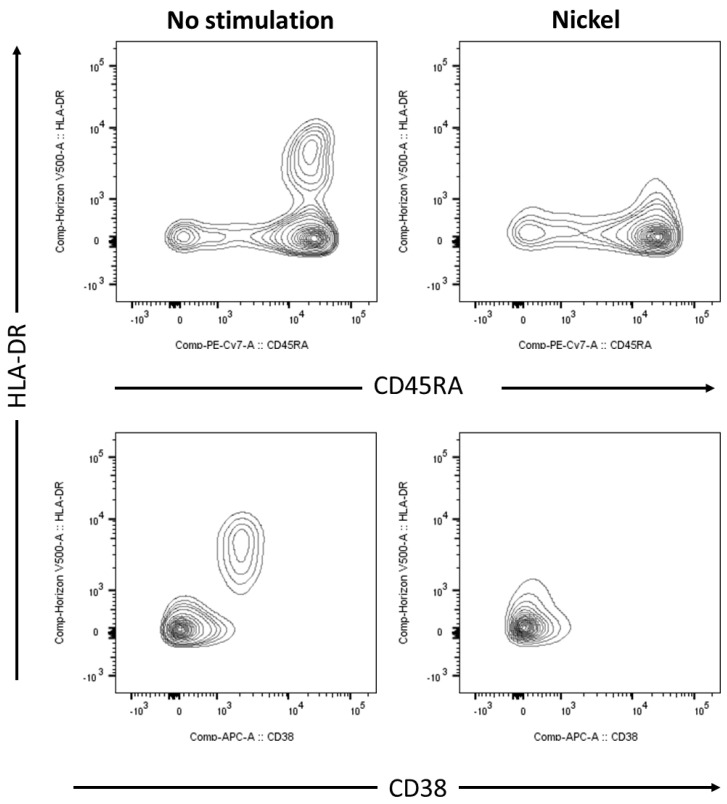
Expression of HLA-DR and CD38 on nickel re-stimulated cytotoxic T cells from blood of patients with nickel allergy. Flow cytometric analyses of PBMCs from human peripheral blood, stimulated for 48 h by 200 µM NiSO_4_ (**right** panel) or control (**left** panel). Total population of PBMCs was gated on viable (L2310 negative), CD3^+^CD8^+^CCR7^+^ cells and were further analyzed for co-expression of HLA-DR and CD38 using a T cell panel as described in materials and methods. FACS analyses show contour plots of one donor, representing results from a total of n = 4 patients suffering from nickel allergy; PBMC, peripheral blood mononuclear cells, NiSO_4_, nickel(II)sulfate.

Using combinatory gating, CD8^+^CCR7^+^ T cells were further analysed for expression of activation marker HLA-DR and CD38 (Figure 2). In untreated cells, a specific subpopulation of HLA-DR^+^cells was identified expressing CD45RA and CD38. Interestingly, if PBMCs from allergic individuals were stimulated by nickel in cell culture, these CD45^+^HLA-DR^+^cells and CD38^+^HLA-DR^+^cells were nearly completely decreased as a distinct population. In contrast, if further analysing CD8^+^CCR7^−^ T cells, effector (CD45RA^+^) and effector memory (CD45RA^−^) populations remained unaffected with respect to weak HLA-DR and CD38 expression after stimulation with nickel.

### 2.3. CD38 Decreased in Cytotoxic T Cells from Patients with Nickel Allergy after Challenge

**Figure 3:**
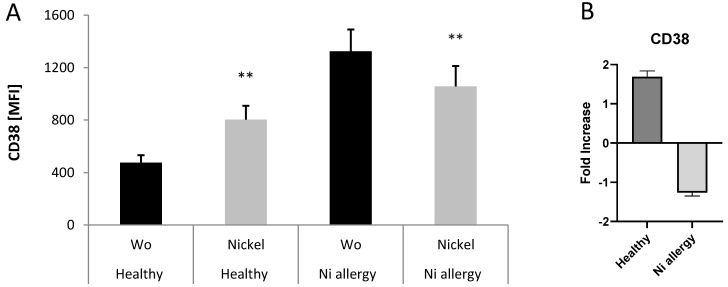
Expression of CD38 on nickel re-stimulated CD3^+^CD8^+^CCR7^+^ T cells from blood of healthy donors and patients suffering from nickel allergy (**A**). Bars represent mean values + SD of mean fluorescence intensities (MFI) for CD38 measurement on PBMCs from three healthy donors and four patients suffering from nickel allergy. PBMCs from both groups were stimulated for 48 h by 200 µM NiSO_4_ or left untreated (Wo) and analyzed using T cell panel as described in materials and methods. Differences of untreated cells to cells stimulated by nickel were analyzed for statistical significance (**, 0.001 < *p* < 0.01) in both groups. Bars in (**B**) indicate analyses of the means for fold increase CD38 in nickel treated cells comparing to unstimulated cells in healthy donors and patients with Ni allergy.

If comparing expression of CD38 (mean fluorescence intensities, MFI) in CD8^+^CCR7^+^ T cells of patients suffering from nickel allergy and CD38 in healthy donors, a specific expression pattern was found (Figure 3A,B). In people without allergy, expression level of CD38 was low and only slightly enhanced by stimulation with nickel in cell culture. Vice versa, in patients with a nickel allergy background, expression level of CD38 was higher and decreased by stimulation with nickel in cell culture. Thus, data from immunophenotyping indicates that activation marker could be increased in subpopulations of T cells derived from the blood of ACD patients. However, nickel-specific activation level could be down-regulated after challenge with the same chemical. 

### 2.4. HLA-DR Increased in T Helper 1 from Patients with Nickel Allergy after Challenge

**Figure 4:**
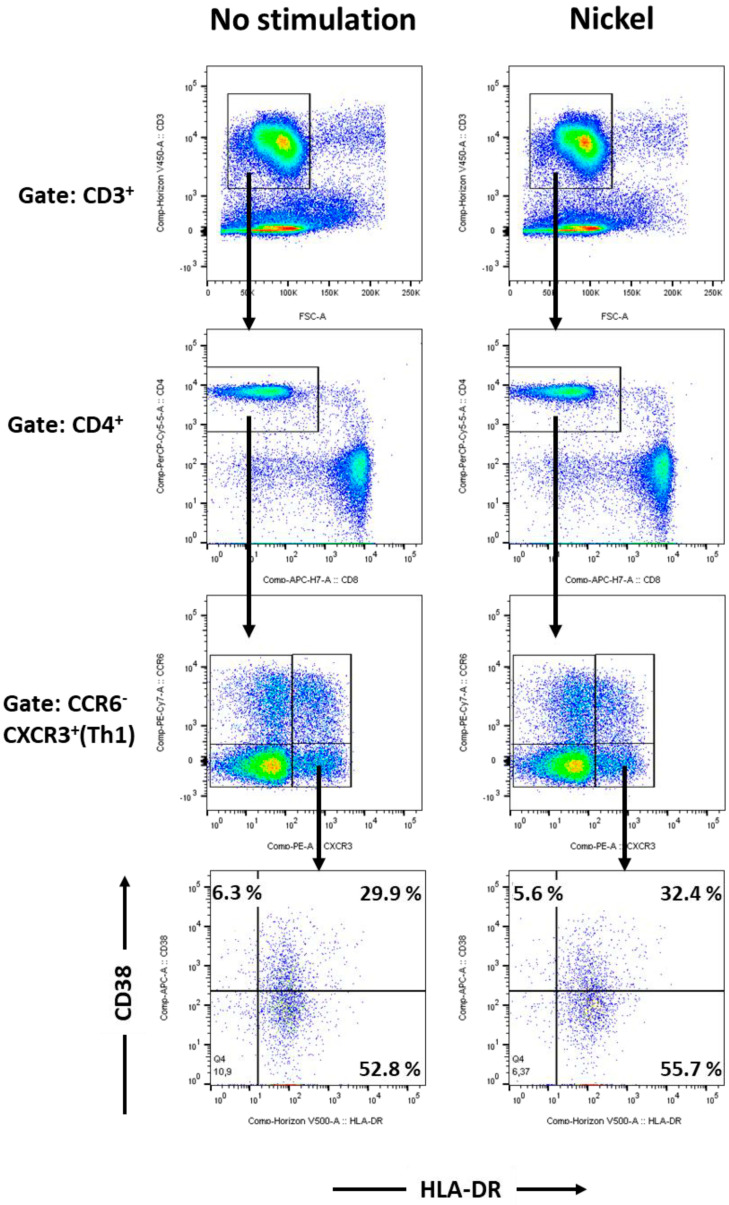
T helper 1 cells: Gating strategy and immunophenotyping of nickel re-stimulated PBMCs from patients suffering from nickel allergy. Flow cytometric analyses of PBMCs from human peripheral blood, stimulated for 48 h by 200 µM NiSO_4_ (**right** panel) or control (**left** panel). T helper cells were stained using a Th1/Th2/Th17 panel as described in materials and methods and discriminated by CD3 (**upper** row), CD4 vs CD8 (**middle** row) and chemokine receptors CCR6 and CXCR3. T helper 1 cells were identified as CD3^+^CD4^+^CCR6^−^CXCR3^+^ cells and analysed for CD38 and HLA-DR expression. FACS analyses show dot plots of one donor, representing results from a total of n = 4 patients suffering from nickel allergy; PBMC, peripheral blood mononuclear cells, NiSO_4_, nickel(II)sulfate.

If investigating T helper cells (Figure 4), individual subpopulations were discriminated by expression of specific chemokine receptors. CD4^+^CCR7^+^CD45RA^+^CCR6^−^CXCR3^+^ were assigned to represent Th1 cells, CD4^+^CCR7^+^CD45RA^+^CCR6^+^CXCR3^−^ to Th17 and CD4^+^CCR7^+^CD45RA^+^CCR6^−^CXCR3^−^ to Th2 cells. In Th1 cells from patients suffering from nickel allergy, HLA-DR increased more than 5% after challenge with nickel. In Th2 and Th17, HLA-DR expression was not affected.

### 2.5. Th1 and Th17-Specific Transcription Factors T-bet and RORγt Increased by Nickel Re-Stimulation

**Figure 5:**
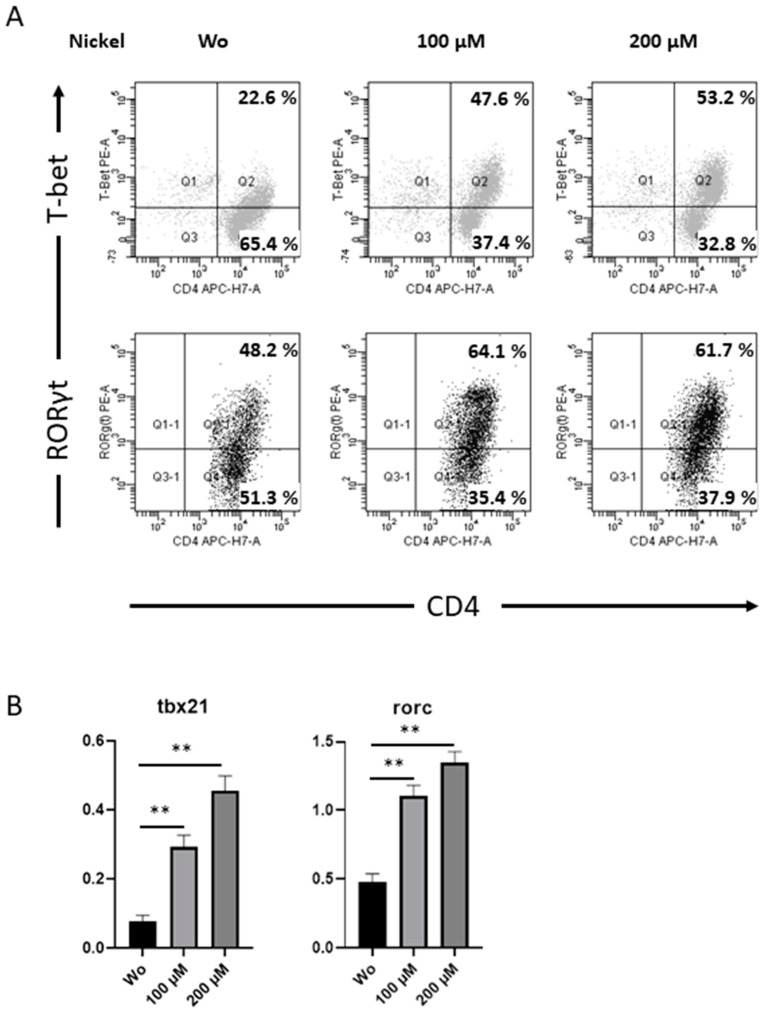
Expression of Th-specific transcriptions factors in viable CD4^+^ Th cells. T helper cells, isolated from PBMCs of human peripheral blood, stimulated for 48 h by 200 µM NiSO_4_ (**right** panel) or control (**left** panel) were co-stained with mAbs against T-bet and RORγt. Intracellular FACS analyses show dot plots of one donor, representing results from a total of n = 4 patients suffering from nickel allergy (**A**). mRNA expression for *tbx21* and *rorc* was measured for donors as in (**A**) by quantitative PCR. Analysis was calculated after normalization to β-actin (**B**); PBMC, peripheral blood mononuclear cells, NiSO_4_, nickel(II)sulfate (**, 0.001 < *p* < 0.01).

To further investigate the effect of nickel on the total population of Th cell and differentiation after challenge, viable CD4^+^ T cells were intracellularly stained for expression of T-bet and RORγt (Figure 5A). Comparing to vehicle treated CD4^+^ T cells, Th cells re-stimulated by nickel showed higher expression of and more cells were found positive for T-bet and RORγt, thereby indicating the differentiation of Th 1 and Th17 cells. These results evaluated on protein level were further confirmed by analyses of transcripts for *tbx21* and rorc using RT-PCR (Figure 5B).

### 2.6. CD25 Increased in CD3^+^CD4^+^ T Cells in Patients with Nickel Allergy after Challenge

**Figure 6:**
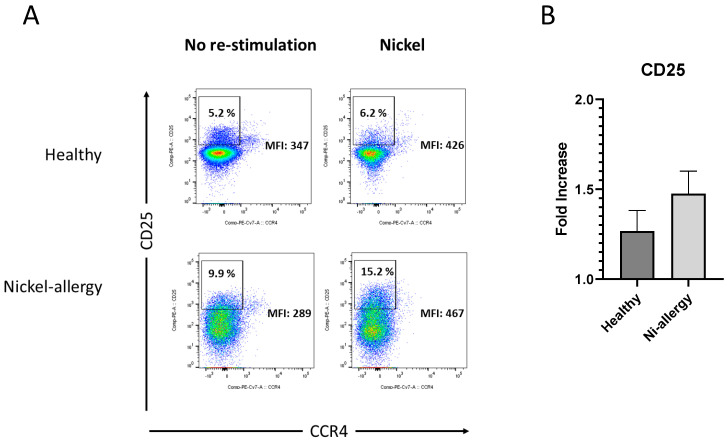
Activated T cells: Gating strategy and immunophenotyping of nickel re-stimulated PBMCs from patients suffering from nickel allergy. Flow cytometric analyses of PBMCs from human peripheral blood (**A**), stimulated for 48 h by 200 µM NiSO_4_ (**right** panel) or control (**left** panel). Th cells were stained using a T reg panel as described in materials and methods and discriminated by gates on viable CD3^+^, CD4^+^ and 127 low cells. FACS analyses show expression of CD25 vs CCR4 of one donor, representing results from a total of n = 4 patients suffering from nickel allergy and n = 3 healthy donors. Bars in (**B**) indicate analyses of the means for fold increase CD25 in nickel treated cells comparing to unstimulated cells in healthy donors and patients with Ni allergy; PBMC, peripheral blood mononuclear cells, NiSO_4_, nickel(II)sulfate.

When analyzing the expression of the marker for activation of Th cells and regulatory T cells (T_reg_), CD25, two findings were recorded (Figure 6). In both groups with and without nickel allergy, CD25 expression significantly increased as observed by upregulation in % positive cells and MFIs after stimulation with nickel in cell culture. This effect was, however, more prominent, but not significantly higher, in patients suffering from nickel allergy than in normal donors. As CD3^+^CD4^+^ cells were negative for CCR4, they were not regarded as T_reg_ but as activated Th cells.

## 3. Discussion

In this study, principal phenotypic characteristics of T cell populations responding to re-stimulation with nickel in cell culture were investigated. In nickel-allergic subjects, biomarkers for cell activation, CD38 and HLA-DR, were found to be affected by nickel-stimulation. In diagnosed people free of nickel allergy, CD38 and HLA-DR were increased by nickel activation in vitro. Enhanced expression was also detected in Th1 cells of the phenotype CD4^+^CCR7^+^CD45RA^+^ CCR6^−^CXCR3^+^ from patients with nickel allergy. Interestingly, in cytotoxic T cells of the phenotype CD8^+^CD45RA^+^CCR7^+^ which could be regarded as naïve T cells decreased expression of CD38 and HLA-DR was observed. In the literature there were only few studies that report on extensive phenotypic characteristics of human T cell subpopulations in individuals with nickel allergy. Effects in naïve CD8^+^ T cells were also reported by Wicks et al. [24]. The group found in the CD8 high expressing population of allergic subjects an increased proportion of naïve cells (CD27^+^ CD45RA^+^), reductions in effector memory (CD27^−^ CD45RA^−^) and decrease the effector memory cells re-expressing CD45RA (CD27^−^CD45RA^+^) subpopulations as compared to healthy controls.

An advantage of allergy studies using PBMCs is the fact that they already include blood dendritic cells (DC) or their precursors as potential antigen-presenting cells. Bechara et al. [25] show that IFN-γ release of CD4^+^ and CD8^+^ T cells was directly dependent on the presence of autologous DCs that were stimulated by 500 µM nickel sulphate. They also studied naïve T cells derived from normal blood of healthy people after nickel exposure. The cells investigated were of the phenotype CD4^+^CD45RA^+^CCR7^+^ and CD8^+^CD45RA^+^CCR7^+^. In this study, the release of cytokine IFN-γ was associated with access towards MHC class II (DP, DQ and DR) and MHC class I (A, B, and C) molecules, respectively. They conclude that CD4^+^ and CD8^+^ T cells with regard to IFN-γ were activated by nickel exposure. One could speculate if naïve CD8^+^ cells after first exposure to nickel differ from CD8^+^ cells after challenge to nickel in reciprocal expression pattern of activation marker. Bechara et al. [25] and we found up-regulation of the markers IFN-γ and CD38 by nickel in healthy people. *Vice versa*, we observed a decrease for CD38 by nickel in patients suffering from nickel allergy. However, further studies that specifically distinguish sensitization and elicitation phase comparing allergic and non-allergic subjects by measurement of further T cell markers might help to shed light on this issue.

In a recent study of an advanced in vitro lymphocyte proliferation test, a stimulated phenotype in memory Th cells was found after treatment with nickel chloride (NiCl_2_) of PBMCs from patients with allergy [26]. The group detected significant increase in the relative frequency of cutaneous lymphocyte-associated antigen, CLA. In contrast, increase was not observed for CCR6 or CRTH2 in Ni-specific memory Th cells in individuals with nickel allergy comparing to healthy individuals. In this study, the population of interest was gated on CFSE low, CD3, CD45RO and CD4, thereby gated on non-proliferating memory Th cells. This data on Th cells complements to results of our study as we found slight increase in frequencies of CD38 and HLA-DR co-positive cells in CD4^+^CCR7^+^CD45RA^+^CCR6^−^CXCR3^+^ Th1 cells. In addition, decrease of CD38 and HLA-DR was observed in naive cytotoxic CD3^+^CD8^+^CD45RA^+^CCR7^+^cells T cells. Taken together, nickel-stimulation of PBMCs from patients with allergy could technically be verified by different studies using flow cytometry and specific surface markers for naive and memory cytotoxic and Th cells. 

There may be a couple of important applications for this new method such as confirmation of patch test results which are reported to sometimes fail a diagnostic clear-cut. In addition, the patch test is a rather qualitative test method, biased by possible different estimations of the findings in optical inspection by the individual physician. Another field of possible application that could benefit from allergic phenotyping is of course regulatory testing. For risk assessment of pesticides, biocides, cosmetics and REACH chemicals, patch testing is for ethical reasons not justified. There were great advances in developing and validation of new in vitro methods within the AOP Skin Sensitization for key events 1–3 such as the human cell line activation Test, h-CLAT, and the ARE-Nrf2 Luciferase Test Method (OECD TG442E and D). The development of a functional T cell assay is a matter of global activities in the regulatory field, with preference in the OECD’s AOP project. But until now in this AOP Skin Sensitization for the final and obviously most specific and predictive key event, T cell differentiation, appropriate testing methods are under development but until now far from validation. In addition, assays have been proposed recently that verify the applicability of human T cell lines to predict a substance’s potential for skin sensitization. In Jurkat E6-1 cells, the early T cell activation marker CD69 was measured by flow cytometry after stimulation by 34 different sensitizers [27]. For nickel chloride and nickel sulfate, CD69 on these T lymphoblasts was demonstrated to reliable sense an allergic potential. However, the absence of APCs in this model is an advantage in laboratory handling but raises the concern for the use with substances that need metabolic activation to become complete allergens. Recently, our group reported on a sensitization assay comprising lymphocytes in addition to keratinocytes and generated DCs. Further lymphocyte markers CD44, CD119, CD124 (IL-4 receptor), and IL-23R were investigated in a coculture system using freshly isolated PBMCs seeded onto primary keratinocytes and enriched for DCs (loose-fit coculture-based sensitization assay) [27]. In this protocol, higher expression of the memory marker CD44 and of CD124 was observed, but nickel was not involved in the study. Clouet et al. [28] proposed a modified h-CLAT protocol which includes allogeneic CD4^+^ T-lymphocytes stained by CFSE. Here, nickel induced about 20% more proliferating cells than vehicle controls.

In our study the marker for activated T cells and T_reg_, CD25, was significantly increased in patients suffering from nickel allergy after re-stimulation by nickel, even more prominent than in healthy controls (Figure 6). We could not deduce an effect in T_regs_ as CD3^+^CD4^+^ cells were negative for CCR4. However, Cavani [29] observed that blood derived CD4 T cells from healthy people showed a restricted potential to proliferate after stimulation with nickel in vitro. Interestingly, T cell proliferation was restored in the absence of CD4^+^CD25^+^ T cells. These cells were further identified as T_regs_ by staining of CLA. Thus, they conclude that T_regs_ could maintain immune tolerance to nickel in non-allergic individuals. The question if T_regs_ play a distinctive role in the control of individual T cell subpopulations also in people with nickel allergy was addressed by Wicks et al. [24]. They demonstrate that CD25^+^ FoxP3^+^ T_regs_ decrease in the group of individuals with rather extreme allergy.

In the present study, we observed an increase for the expression of nuclear receptors T-bet and RORγt in Th cells from human PBMCs. There were only few reports investigating transcription factors as candidates for biomarkers in sensitization assays. Previously, T-bet and GATA-3 were used in DC/T cell based immunoassays for detection of respiratory sensitizers [30]. In this coculture system, the chemical OPA induced mRNA upregulation of Th2 specific GATA-3 in allogeneic naive CD4^+^ T cells. However, increase of T-bet and also c-Fos on the transcription level was not significant. In that study, expression of the transcription factors c-Fos, T-bet, and GATA-3 was combined with analysis of their respective target cytokines IL-2, IFN-γ, and IL-4. Until now, one can only speculate, if detection of transcription factors in Th cell subpopulations received by the application of a specific gating strategy in our and the protocol used in study by Mizoguchi [30] could enhance sensitivity and specificity in immune-cell based sensitization assays.

Thus, there were multiple indications from our and further studies that detection of Th cell transcription factors on transcript and protein level could be suited for application in sensitization assays. If transcription factor expression in Th cells from patients suffering from nickel allergy is affected and could be used as a reliable allergy marker as well remains to be investigated by further studies.

## 4. Materials and Methods

### 4.1. Blood Donors and Patch Testing

All studies were performed with ethical approval EA1/201/09 of the department of dermatology, Allergy Center Charité. All participants provided their written informed consent to donate their blood for research purposes. Anonymized blood samples of healthy donors were obtained from the German Red Cross blood donation service Berlin with informed written consent from all participants. All studies have been approved by the Institutional Review Board and were in accordance to the Helsinki guidelines. Peripheral blood samples of volunteers confirming their informed consent were used for nickel re-stimulation experiments and FACS analyses. Four donors showed a clinical history of contact allergy and were positively patch tested for nickel sulfate at the beginning of the study, three people were negatively patch tested for nickel sulfate and served as controls. Patch testing followed international recommendations [23].

### 4.2. White Blood Cell Preparation, Cell Culture and Stimulation with Nickel

PBMCs were prepared from samples of patients suffering from nickel allergy and from buffy coats of healthy adult donors by Ficoll gradient (PAA Laboratories, Pasching, Austria). In some experiments, T helper cells were further enriched from PBMCs by depletion of different cell populations using the MACS system (CD4^+^ T Cell Isolation Kit, Miltenyi Biotec, Bergisch Gladbach, Germany). Isolated cells were plated in 24-well (PBMCs) or 12 -well (CD4^+^ T cells) flat botton multiwell culture dishes (Greiner, Kremsmünster, Austria) at a cell number of 10^6^ cells per ml. All cells were kept in RPMI 1640 supplemented with 2 mM L-glutamine, 100 U/mL penicillin, 100 mg/mL streptomycin, and 10% *v*/*v* heat-inactivated FBS (PAN Biotech, Aidenbach, Germany). For protein and PCR analyses the total population of PBMCs or where indicated, T helper cells, were stimulated by 200 µM nickel(II)sulfate (NiSO_4_) (Sigma Aldrich, St. Louis, MO, USA) for 12 and 48 h, respectively. Preparations of NiSO_4_ contained endotoxin below 0.1 EU/mg compound, as determined by using the Limulus amebocyte lysate assay (BioWhittaker, Walkersville, MD, USA).

### 4.3. Staining for Phenotyping and Antibodies Used

Antibodies were selected for isotype and fluorochrome label according to the standard protocol for phenotyping of human PBMCs, published by the Stanford Human Immune Monitoring Center ^6^. In premixed plates the following optimized antibody panels were used. T cell panel: CD3 (Horizon V 450), CD4 (PerCP-Cy5.5), CD8 (APC-H7), HLA-DR (Horizon V500), CD45 RA (PE-Cy7), CCR7 (PE), CD38 (APC); Th1/Th2/Th17 panel: CD3 (Horizon V 450), CD4 (PerCP-Cy5.5), CD8 (APC-H7), HLA-DR (Horizon V500), CCR6 (PE-Cy7), CXCR3 (PE), CD38 (APC); T reg panel: CD3 (Horizon V 450), CD4 (PerCP-Cy5.5), CCR4 (PE-Cy7), CD25 (PE), CD127 (APC) and isotype controls with corresponding fluorochromes. The follow-up protocol was kindly provided by bd biosciences. Transcription factors were co-stained intracellularly by anti-T-bet (PE, 4B10) or anti-RORγt (PE, AFKJS-9), both purchased by eBioscience. Debris and doublets were excluded by scatter and viability was confirmed by co-staining using Live/Dead Green dye (L23101, Invitrogen). Cells were analysed in the FACS LSR II (BD Biosciences, Heidelberg, Germany) flow cytometer using FACS-Diva and FlowJo software.

### 4.4. PCR Analysis

Total RNA was extracted from MACS enriched CD4^+^ T cells using RNeasy kits (Qiagen, Hilden, Germany), cDNA was synthesized using PCR oligo(dT) primer pairs provided by Thermo Fisher and SuperScript III (Invitrogen, Waltham, MA, USA) according to the manufacturer’s instructions. The transcription level of *tbx21* and rorc were assessed by quantitative real-time PCR using an ABI Prism 7500 Real-Time PCR system (Applied Biosystems). β-Actin was used to normalize the cycle threshold value.

### 4.5. Statistical Analysis

A Student’s paired *t* test was used to calculate statistical significance. *p*-Values below 0.01 were considered significant. Calculations were computed with the statistics tools provided by R (R-project.org).

## Data Availability

Data not available due to ethical restrictions.
